# Coordinates and intervals in graph-based reference genomes

**DOI:** 10.1186/s12859-017-1678-9

**Published:** 2017-05-18

**Authors:** Knut D. Rand, Ivar Grytten, Alexander J. Nederbragt, Geir O. Storvik, Ingrid K. Glad, Geir K. Sandve

**Affiliations:** 1 Department of Mathematics, University of Oslo, Moltke Moes vei 35, Oslo, 0851 Norway; 2 Department of informatics, University of Oslo, Gaustadalleen 23 B, Oslo, 0371 Norway; 3 Department of Biosciences, University of Oslo, Blindernvn. 31, Oslo, 0371 Norway

**Keywords:** Pan-genome, Sequence graphs, Reference genome, Epigenomics

## Abstract

**Background:**

It has been proposed that future reference genomes should be graph structures in order to better represent the sequence diversity present in a species. However, there is currently no standard method to represent genomic intervals, such as the positions of genes or transcription factor binding sites, on graph-based reference genomes.

**Results:**

We formalize offset-based coordinate systems on graph-based reference genomes and introduce methods for representing intervals on these reference structures. We show the advantage of our methods by representing genes on a graph-based representation of the newest assembly of the human genome (*GRCh38*) and its alternative loci for regions that are highly variable.

**Conclusion:**

More complex reference genomes, containing alternative loci, require methods to represent genomic data on these structures. Our proposed notation for genomic intervals makes it possible to fully utilize the alternative loci of the GRCh38 assembly and potential future graph-based reference genomes. We have made a Python package for representing such intervals on offset-based coordinate systems, available at https://github.com/uio-cels/offsetbasedgraph. An interactive web-tool using this Python package to visualize genes on a graph created from GRCh38 is available at https://github.com/uio-cels/genomicgraphcoords.

**Electronic supplementary material:**

The online version of this article (doi:10.1186/s12859-017-1678-9) contains supplementary material, which is available to authorized users.

## Background

A reference genome for a species makes it possible to represent genomic features from different sources in a common reference. Examples of such features can be genes, methylation status or histone modifications. The common reference enables analyses of the relationship between these features, e.g. computing the distance between a gene and a regulatory element. In order to perform such computations generically, a common coordinate system on the reference genome is needed.

Formally, a *reference genome coordinate system* is a system that uses coordinates to uniquely determine the positions of bases in the reference genome. Until recently reference genomes have exclusively been represented in linear form, meaning that there is only one path from the beginning of each chromosome (or sequence element) to the end. This enables a coordinate system where each base can be uniquely identied by the chromosome ID and the offset from the beginning of the chromosome. An example of a coordinate is chr14, offset 150,000,000, written more compactly as chr14:150m. A genomic interval can then be represented unambiguously by two such coordinates, the start and end position of the interval. Such a linear representation simplifies genomic arithmetics, done by e.g. *bedtools* [1] and the *GSuite Hyperbrowser* [2].

A problem with linear reference genomes is that they are unable to represent variation within a species, making them incapable of adequately representing features from individuals that are very different from the reference. A solution to this problem is to use *sequence graphs* as reference structures [3–6].

**Fig. 1:**
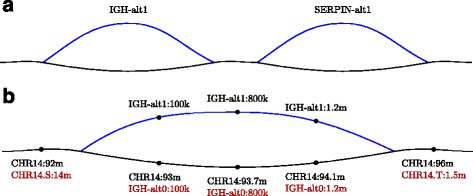
Coordinates in GRCH38. **a** Visualization of alternative loci in the IGH and the SERPIN region on chromosome 14 in GRCh38. The main path is shown in *black* and an alternative locus in *blue*. **b** A closer look at coordinates in the IGH region, with coordinates obtained from hierarchical partitioning (*black*) and sequential partitioning (*red*). On the alternative locus, the coordinates are the same, as this is a separate region path in both partitionings. Using the sequential partitioning, coordinates on the main path are more similar to those on the alternative loci

In addition to the 24 primary chromosome assemblies, the newest human reference genome, Genome Reference Consortium Human Build 38 (GRCh38), contains 261 alternative loci, regions with significantly different sequences between individuals. GRCh38 can be represented as a sequence graph by connecting the alternative loci to the primary chromosomes (Fig. 1 a). Generally, a sequence graph can represent genomes from one or more individuals along with additional variation data. Each vertex in the graph represents a DNA-sequence of one or more base pairs, and each edge connects two consecutive sequences in the genome. Examples of the use of sequence graphs are De-Bruijn graphs in *de novo* assembly [7–9], the software project VG [10] that has built a framework for representing variation data on graphs, the *FASTG* [11] and *GFA* [12] formats, as well as in certain applications for genotyping [13] and multiple sequence alignment [14].

Referencing positions and intervals on graph-based reference genomes poses challenges not present with linear reference genomes. First, positions such as chr1:100 will be ambiguous if there are multiple positions on chromosome 1, on different paths, having offset 100. Second, intervals represented by only a start and an end coordinate will be ambiguous, since there can be different paths taken within the interval. For instance, there is no standard method of representing genes that are partly on alternative loci in GRCh38.

In this article we address these issues by discussing suitable coordinate systems and proposing interval representations for graph-based reference genomes, using GRCh38 and its alternative loci as an example.

## Results and discussion

### Coordinate systems on graph-based reference genomes

The newest human reference genome, GRCh38, can be seen as a graph. Defining a coordinate system on such graph-based reference genomes has been discussed by Marschall et al. [4]. They propose that nearby bases should have similar coordinates (here we name this *spatiality*), that coordinates should be concise and interpretable (here named *readability*), and that coordinates should increment along the genome (here named *monotonicity*). In addition, we propose that the coordinate system should be *backward compatible*, meaning that if the reference graph has been updated, coordinates from a previous version of the graph should preferably still be valid and unambiguously refer to the same bases in the new graph.

Spatiality and readability are useful in order to manually check the validity of results obtained from a computer analysis, while backward compatibility removes the need of updating all previous data when updating the reference graph. An update to the reference genome can include various types of alterations to the graph, e.g. edge/vertex removal or vertex merging, but here we will only look at updates in which new paths are added to the graph.

In this article, we discuss a class of coordinate systems which we denote as *offset-based coordinate systems*. In offset-based coordinate systems, coordinates consist of a region identifier and an offset that is counted from the start of the region. Offset-based coordinate systems include those used on linear genomes today, with chromosome IDs as region identifiers and offsets counted from the beginning of each chromosome, e.g chr1:100. This class of coordinate systems has intuitive and readable coordinates, and two coordinates representing bases close to each other in the genome will be similar. Also, computing the distance between two coordinates within the same region is as simple as taking the difference between the offsets.

**Fig. 2:**
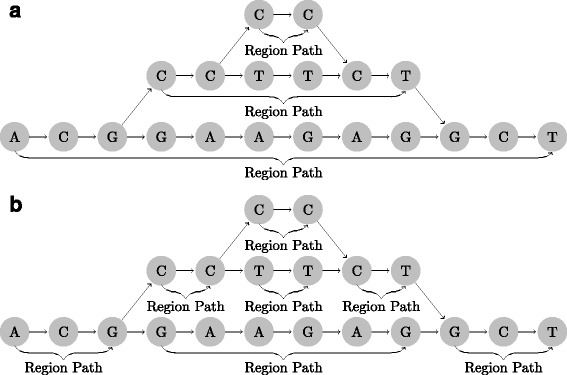
Region path partitions. **a** Hierarchical partitioning. The graph is partitioned into the main region path (*bottom*) and an alternative region path (*middle*) with its own alternative region path (*top*). **b** Sequential partitioning. The graph is partitioned into unambiguous region paths. Both the *top* and *bottom* layer are divided into three region paths

Offset-based coordinate systems can be defined on graph-based reference genomes by partitioning the graph into a set of non-overlapping linear sequences, which we denote as *region paths*. There are different ways to divide the reference genome into region paths, and this is what separates different offset-based coordinate systems from each other. Here we will discuss two variants, which we refer to as *hierarchical* and *sequential partitioning* (Fig. 2).

#### The hierarchical partitioning

The most common way of referencing positions on GRCh38 today is using an offset-based coordinate system. This coordinate system is obtained by what we define as hierarchical partitioning: choosing one main region path through the graph, and defining alternative loci as alternative region paths.

Thus, the offsets for positions on the main region paths are counted from the beginning of each chromosome, and offset for positions on alternative loci are counted from the beginning of each alternative locus. This can be extended to graphs with more layers of alternative loci (e.g. alternative loci of alternative loci) by choosing a main region path for each layer, and defining its alternative loci as region paths (Fig. 2 a).

This coordinate system is backward compatible, since adding a new path to the reference will not change the existing coordinates. However, spatiality is not fulfilled (Fig. 1 b).

#### The sequential partitioning

An alternative to the hierarchical partitioning is to also divide the main paths wherever an alternative path starts or stops (Fig. 2 b, see Additional file 1 for details). Thus, adding a new alternative path to the reference graph will lead to three new region paths on the main path (before, parallel to, and after the alternative path). This change in the partitioning breaks backward compatibility, since coordinates on the old region path are changed when updating the graph. Thus, in order to keep backward compatibility, one needs to keep record of what region path the three new region paths come from, which will make it possible to map coordinates on the old region path to coordinates on the new one.

### Naming schemes

Naming individual region paths is not straightforward, as there are multiple criteria that can be in conflict with each other: The ability to deduce from the names whether two region paths are variants of each other (vertical spatiality), whether they are close to each other along the genome (horizontal spatiality), and which comes first in the genome (monotonicity). Two naming schemes are commonly used in GRCh38: the GenBank sequence accession number (e.g. KI270758.1) [15], and a combination of the region name and the assembly-unit (e.g. MHC ALT_REF_LOCI1). The first naming scheme fulfills none of the above criteria, whereas the second only fulfills vertical spatiality.

Achieving all three criteria can be a challenge when new region paths are added. For instance, when a region path is added between two existing region paths, the name of the new region path should ideally indicate that it is closer to each of the other two region paths than they are to each other, something that can result in less readable region path names after many iterations of changes to the reference genome. In some cases, it is possible that the best solution is a naming scheme not fulfilling all criteria; sacrificing efficiency in updating the reference graph and ease of manually inspecting data for a more lightweight coordinate system.

### Interval representations on graph-based reference genomes

A genomic interval on a graph-based reference genome can either be a single path between two vertices (single-path intervals) or a set of paths between two vertices (multipath intervals). We here propose a method for representing single-path intervals, and present possible methods for representing multipath intervals, on offset-based coordinate systems.

#### Single-path intervals

A natural way to define a genomic interval in a graph-based reference genome is as a single path between two vertices. Such an interval should be represented unambiguously. When using a linear reference structure, the demand for unambiguity can be satisfied by storing the start and end coordinates of the interval. On a graph-based reference genome, this will not give unambiguous intervals if there is more than one path between a start and an end coordinate in the graph.

To resolve this problem, the representation must indicate which path the interval follows in the graph. The minimal solution is to indicate which region paths the interval follows wherever it is ambiguous. An interval can then be represented as a list containing the start and end coordinates in addition to information about the region paths followed by the interval.

An example of a genomic interval represented this way is (chr14:100, IGH-alt1, SERPIN-alt1, chr14:150m), which denotes the interval from offset 100 to 150m on chromosome 14 going through the region path IGH-alt1 and the region path SERPIN-alt1 (Fig. 1 b). This representation does not indicate that the interval follows the main path between the IGH-alt1 and the SERPIN-alt1 region paths, since the path taken here is unambiguous.

**Fig. 3:**
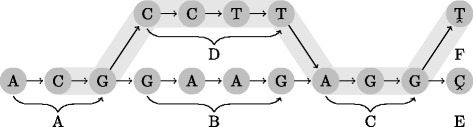
An interval spanning the region paths *A*, *D*, *C* and *F*. Using the minimal notation, this would be represented as (A:1 D F:0), but with the proposed full notation the C region path is also included: (A:1 D C F:0)

However, we argue that also including information about unambiguous paths will improve readability (see Additional file 1). In the above example, this would make it possible to deduce that the interval follows the main path in between the IGH and the SERPIN regions. The interval would then be represented as (chr14:100, IGH-alt1, chr14, SERPIN-alt1, chr14:150m). This representation is equivalent to including a region path identifier every time the identifier changes (Fig. 3). If the coordinate system is backward compatible, intervals defined on the coordinate system will also be backward compatible, since the intervals can be uniquely determined by a set of (backward compatible) coordinates (e.g. the coordinate of the first vertex in each region path).

#### Multipath intervals

The representation of single-path intervals on complex graphs may become unreadable due to the number of region paths that need to be specified. Moreover, the exact path of an interval, e.g. what SNP variations a gene has, may not be of interest. In such cases multipath intervals can be appropriate.

One way of representing multipath intervals is to *explicitly* list all paths included in the interval representation, which will lead to less concise representations on complex graphs. Alternatively, one can only explicitly list the start and end coordinate of the interval, and *implicitly* include all paths between those. While this leads to more concise representations, it cannot represent intervals that only follow a subset of the paths between the start and end vertex. We propose two alternative representations, combining the principles of explicitly representing paths and implicitly inferring paths from the representation, to achieve a better trade-off between expressive power and conciseness.

The *critical subpaths* representation uses a set of *critical subpaths* to denote all paths between the start and end vertex that traverse all the critical paths. Using this representation, one can allow variation in some regions, while being specific in others. For instance, genes can be represented this way by specifying exonic regions as critical paths, and interpret the gene as all paths through these.

**Fig. 4:**
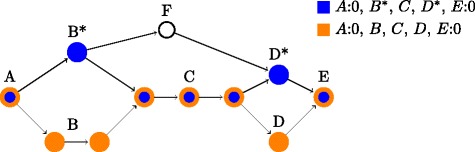
Multipath intervals. Illustration of two genomic features represented by single-path intervals (*orange and blue*). Assuming that which paths are taken between *A* and *E* is irrelevant, as long as they go through *C*, both can be represented by the same critical multipath interval *A*_0_,*E*_0_,*critical*=*C*. The same can be achieved by using a fuzzy interval with either of the two singlepath intervals as a main path and a threshold of 2, since the edit distance between region path *C* and *F* is 3. Note that if using a threshold of 1, both region path *B* and *B* ^∗^ cannot be contained in the same multipath interval

The *fuzzy path* representation uses a central path between the start and end vertex, along with a threshold, to denote all paths deviating from the central path with a distance less than the threshold. Different definitions of the edit distance can be used, for instance global edit distance or the maximum of local edit distances (see Additional file 1). Figure 4 shows an example of different ways to represent two intervals using single- and multipath intervals.

Both these methods have the ability to represent intervals at different levels of resolution, either by specifying more or fewer critical subpaths, or varying the threshold parameter. Large genomic areas, such as the human leukocyte antigen (HLA) super-locus in the human genome, can be represented at low resolution, large elements such as genes can be represented with higher resolution and small elements such as mapped reads can be represented at the highest resolution, accurately representing their exact sequence.

### Example: genes on GRCh38

We here show how our proposed interval representations can be used on graph-based reference genomes, by representing GRCh38 transcript variants from the RefSeq gene database [16] on graphs created from GRCh38. First, we show how the single-path interval representation gives a more natural image of these transcript variants. Second, we show how multipath intervals can be used to represent transcript variants on a more detailed graph.

### Representing transcript variants using single-path intervals

The alternative loci of GRCh38 contain regions at the beginning and end with identical sequence to the main path. These *flanking regions* provide contexts to the non-flanking parts — the *varying regions* of the alternative loci. When creating a graph from GRCh38, these contexts are provided by the connections to the main path, and so the flanking regions are unnecessary. Thus, we create a graph by removing these flanking regions from the alternative loci and connecting only the varying regions of the alternative loci to the main path.

The Refseq database contains 6871 transcript variants on the alternative loci of GRCh38 (*alt-locus transcripts*) and 60348 transcript variants on the main path (*main-path transcript*), each identified by a Refseq transcript identifier. We look at pairs of alt-locus transcripts and main-path transcripts that have the same Refseq transcript identifier, to show how our single-path interval representation can give a clearer image of these pairwise relationships.

**Fig. 5:**
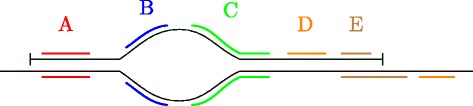
Five types of alt-locus transcripts. Category A (*red*): Alt-locus transcripts on flanking regions that have nearly identical copies on the main path. Category B (*blue*): Alt-locus transcripts on the varying regions that have similar transcripts on the main path. Category C (*green*): Alt-locus transcripts that are partly on flanking regions, and have similar transcripts on the main path. Category D (*orange*): Alt-locus transcripts on the flanking regions that have copies somewhere else on the main path (not parallell to the alt locus). Category E (*brown*): Alt-locus transcripts on flanking regions that have copies on the main path that also extend outside the alternative locus

We represent all Refseq transcripts on the graph created from GRCh38, by representing each transcript as a list of single-path intervals (one interval for each exon). Since some transcript variants (in total 274) span multiple region paths, we need our, or a similar, interval representation to be able to represent them. Also, by using sequential partitioning, it is easy to see whether two transcript variants are on parallel paths in the graph. We categorize all alt-locus transcripts based on their relationship with their corresponding main-path transcript. These categories are illustrated in Fig. 5. Table 1 shows the number of transcripts in each category. These result show that there are alt-locus transcripts that have been unnaturally cut on the alt locus border (category E). Also, eight alt-locus transcripts on the flanking regions do not have a copy on the corresponding part of the main path (category D). It is possible that these alt-loci transcripts should have been removed or that a corresponding main-path transcript should have been included (see Additional file 1 for details).

**Table 1:** Number of alt-locus transcripts from GRCh38 in different categories

	RefSeq alt-locus transcripts
Category A	690
Category B	5007
Category C	198
Category D	8
Category E	223
None of these categories	1522

Categories are described in Fig. 5

### Representing transcript variants using multipath intervals

We also show how multipath intervals can be used to compare alt-loci transcripts with main-path transcripts. To do this, we create a more detailed graph by using the alignments of the alternative loci to the primary assembly generated by NCBI [17] to merge the alternative loci to the main path. This creates a graph where the regions on the alternative loci that have common sequence with the main path are merged to single paths interspersed with diverging paths representing insertions, deletions or SNPs. We represent transcript variants (from RefSeq and GENCODE [18]) on this graph using two different multipath interval representations — critical subpaths and fuzzy path multipath intervals. Table 2 shows the number of alt-loci transcripts that on this graph have an identical representation to a main-path transcript. These results show that by representing transcript variants as multipath intervals, the number of duplicate representations for a transcript variant can be reduced (Additional file 1).

**Table 2:** Multipath gene experiment

	RefSeq	GENCODE
Critical intervals multipath interval	2382	2546
Fuzzy path multipath interval	3577	3431
Number of alt-locus transcripts	6871	7089

Number of alt-locus transcripts that can be represented with the same multipath interval as a main-path transcript

### Software

**Fig. 6:**
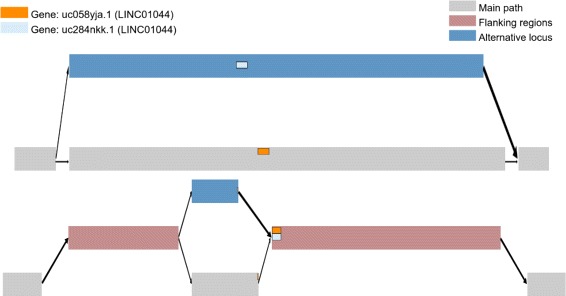
Example from the web tool. Visualization of two sequence-graphs, both around Region 168 on chromosome 13 from GRCh38, created by the web tool. A simple graph with the alternative locus is shown at the *top*. On the *bottom*, a graph created by removing the flanking regions from the alternative locus before connecting it to the main path. Two genes (uc284nkk.1 on the main path and uc058yja.1 on the alternative locus) are shown. On the *top* sequence graph, these genes are on separate paths. By removing the flanking regions and using our notation for genomic intervals (*bottom graph*), we see that uc284nkk.1 and uc058yja.1 are very similar. Both end at the same position within a flanking region, and, barely visible from the figure, they have approximately 1000 base pairs each at the beginning that are different

We have created a Python package, *OffsetBasedGraph*, that can be used to represent intervals on offset-based graphs, available at https://github.com/uio-cels/offsetbasedgraph. An interactive web-tool using this Python package to visualize transcript variants on a graph created from GRCh38 can be found at https://github.com/uio-cels/genomicgraphcoords (Fig. 6), along with instructions for running the gene experiments presented in this section. Additional file 2 shows an example of the notation for transcript variants from GRCh38.

## Conclusion

After more than three years since the release of GRCh38, only a few bioinformatic tools are using its alternative loci, viz. BWA-MEM [19], iBWA [20], GSNAP [21], and SRPRISM [22]. In order to realize the potential of this additional data in GRCh38, one needs a common framework for referencing positions and intervals in the reference genome. An offset-based coordinate system makes it possible to reference positions, but there is currently no standard approach to reference intervals in graph-based coordinate systems.

We propose a simple way to unambiguously represent genomic intervals by including information about all region paths covered by the interval, as well as the start and end coordinates, using an offset-based coordinate system. We also present ways of representing multipath intervals, and show how these can be used to represent genes on a graph created from GRCh38. Being able to represent genomic intervals on graph-based reference genomes makes it possible, for instance, to analyse a gene on an alternative locus in GRCh38 with epigenetic data (e.g. methylation status) from the main path.

Working with graph-based genomes will inevitably lead to complications not present with linear reference genomes. While the coordinate system for linear reference genomes is simple and achieves our discussed criteria (spatiality, readability, monotonicity and backward compatibility), a graph-based coordinate system will be much more complex and only partially meet these criteria. Thus it is necessary to weigh the different criteria, as well as the overall goal of simplicity, against each other in order to find the most suitable coordinate system.

With a system for representing genomic intervals on graph-based reference genomes and a common coordinate system, researchers can begin to utilize more fully the potential of GRCh38 and future graph-based reference genomes.

## Methods

Graphs were created using positions of GRCh38 alternative loci collected from NCBI [23]. The detailed graphs were created using alignments of the alternative loci to the primary assembly generated by NCBI [17]. In all cases, sequence data obtained through the UCSC DAS server were used to merge regions with sequence identity between the main chromosomes and alternative loci.

Gene locations from Refseq [16] and Gencode [18] were downloaded using the UCSC Table Browser [24] and translated to the graph coordinate systems. Both graph creation and interval translation were performed using the software package *Offsetbasedgraph*. The exact data used is available in the supporting Github repository at https://github.com/uio-cels/genomicgraphcoords.

The software package *Offsetbasedgraph* is implemented using Python, and the web tool uses Javascript and HTML to visualize gene positions on alternative loci.

## Additional files

Additional file 1 Definitions and experiment details. Formal definitions of terms used in the article and details on the section Genes on GRCh38 (pdf). (PDF 181 kb)

Additional file 2 Example of gene notation. Example of representation of genes on an alternative locus on GRCh38 (txt). (TXT 4 kb)

## Abbreviations

*GRCh38*: Genome Reference Consortium human 38
