# Supplementary material for: Coordinates and intervals in graph-based reference genomes
Source: BMC Bioinformatics. 2017 May 18;18:263. doi: 10.1186/s12859-017-1678-9 (PMC5437615; doi:10.1186/s12859-017-1678-9)
Supplement: Supplementary file 1 — Definitions and experiment details. Formal definitions of terms used in the article and details on the section Genes on GRCh38 (pdf). (PDF 181 kb) [file 12859_2017_1678_MOESM1_ESM.pdf]

Additional File 1

May 2, 2017

Contents

1 Definitions **1**
1.1 Graphs 1
1.2 Sequence graphs 2
1.3 Region Path Partitioning 2
1.4 Offset-based Coordinate Systems 3
1.5 Single-path Intervals 3
1.6 Multipath Intervals 3

2 Experiments **4**
2.1 Relationship between transcripts on alternative loci and the main path of GRCh38 5
2.2 Representing transcripts using multipath intervals 6

1 Definitions

1.1 Graphs

A directed graph $G = (V, E)$, later only called a *graph*, is a set of vertices V and a set of edges E where each $e \in E$ is an ordered tuple (v_1, v_2) where $v_1, v_2 \in V$. For a graph $G = (V, E)$ we use the following definitions:

- A *walk* of *length* n is a sequence of alternating vertices and edges $(v_0, e_1, v_1, e_2 \dots e_n, v_n)$ such that $e_i = (v_{i-1}, v_i) \in E$ for each $i \in \{1, 2, \dots, n\}$.
- A *path* is a walk where no edge or node, except possibly the first and last, is repeated.
- The *distance* $\text{dist}(v_1, v_2)$ between two vertices v_1 and v_2 is the length of the shortest path between the vertices.
- G is *connected* if every pair of vertices $v_1, v_2 \in V$ is connected by a walk.
- G is *acyclic* if there does not exists a walk of length larger than 1, that starts and ends at the same vertex.
- A graph $H = (V', E')$ is a *subgraph* of G if $V' \subset V$ and $E' \subset E$

1.2 Sequence graphs

We look at graphs where each vertex represents a base pair. A sequence S of base pairs, represented by letters in the finite alphabet $\alpha = \{A, G, C, T\}$, can be represented as a graph by letting the base pairs be vertices and have edges between consecutive base pairs. A set of sequences can also be represented as a graph in the same way, but such graphs might not be connected. By including information about how the sequences are connected, they can be connected in the graph by adding edges.

This can be done with a reference genome such as GRCh38, in which there is a sequence for each chromosome and each alternative loci, and there is information about where the alternative loci are connected with the main path sequences.

1.3 Region Path Partitioning

We let a *region path partitioning* $P = \{P_1, P_2, \ldots, P_N\}$ of a sequence graph $G = (V, E)$ be a partition such that:

- $\cup_{i=1}^{N} P_i = V$
- $P_i \cap P_j = \emptyset \quad \forall i, j$
- $\emptyset \notin P$
- The induced subgraph $G_p = (P, E^p) = (p, \{(v_1, v_2) \in E : v_1, v_2 \in p\})$ for each region path $p \in P$ is an acyclic connected subgraph where each $\max_{v \in p} \text{outdegree}(v) \leq 1$ and $\max_{v \in p} \text{indegree}(v) \leq 1$.

We look at two region path partitionings, *hierarchical* and *sequential*. The hierarchical partitioning is obtained by, starting with the complete graph G_0 and level $L = 0$, recursively:

- Choosing one path between two leaf nodes in the graph, and save this as a region path $P_i = \{v_{i1}, v_{i2}, v_{i3}, \ldots, v_{iN}\}$
- The graph $G_i = (V \backslash P_i, \{(v_1, v_2) \in E : v_1, v_2 \notin P_i\})$ is divided into maximally connected subgraphs $SG_1 \cup SG_2, \cup \ldots \cup SG_M$.
- Repeat for each subgraph SG_i on layer $L + 1$

In order to enable unambiguous interval representation, care must be taken in step two of this process so that no two region paths have two edges in the same direction between them and that no region paths have an edge to itself. This can be avoided by either splitting region paths where necessary, or by adding empty region paths that split edges that leads to ambiguity. The latter alternative has the advantage of maintaining backwards compatibility when ambiguity is introduced in an update to the graph.

The *sequential partitioning* is obtained by dividing the graph near every vertex v where either indegree(v) or outdegree(v) is higher than 1. Vertices

with indegree(v) > 1 are included in the following region path, while those with outdegree(v) > 1 are included in the preceding region path. Thus a vertex that has both indegree(v) > 1 and outdegree(v) > 1 is its region path's only vertex.

The sequential partitioning is unique for a graph, while there can be several possible hierarchical partitionings for a graph.

1.4 Offset-based Coordinate Systems

Let regpath(v) of a vertex v be the unique region path that v is a part of, and $v_0(R)$ of a region path R be its first vertex. Using a region path partitioning P, one can then define a coordinate system on the graph using the functions regpath(v) and and $\text{offset}(v) = \text{dist}(v, v_0(\text{regpath}(v)))$. The coordinates for a vertex is then $(\text{regpath}(v), \text{offset}(v))$, uniquely identifying each vertex in the graph.

1.5 Single-path Intervals

We define a *single-path interval* on a graph as a path $p = (v_0, e_1, v_1, e_2, \ldots e_n, v_n)$ between two vertices v_0, v_n. In order to uniquely represent a path p unambiguously, information about which edge is followed is required wherever outdegree(v_i) $>$ 1. This information can be included as v_{i+1}, since $e_{i+1} = (v_i, v_{i+1})$. Thus, if $V_{\text{split}} = \{v \in \{v_0, v_1, \ldots, v_n\} : \text{outdegree}(v) > 1\}$ the path can be represented by the tuple $(v_0, \{v_i \in p : v_{i-1} \in V_{\text{split}}\}, v_n)$.

One can also use region path identifiers as information about the path. $(v_0, \{\text{regpath}(v_i) \in p : v_{i-1} \in V_{\text{split}}\}, v_n)$, and if using an offset based coordinate system, one can use these coordinates for the start and end vertex.

$$
\begin{aligned}
&((\text{regpath}(v_0), \text{offset}(v_0)),\\
&\ \{\text{regpath}(v_i) \in p : v_{i-1} \in V_{\text{split}}\}),\\
&\ (\text{regpath}(v_n), \text{offset}(v_n))
\end{aligned}
$$

By extending the representation to also including vertices with indegree(v) $>$ 1, readablilty can be improved. Using the set $V_{\text{merge}} = \{v \in \{v_0, v_1, \ldots, v_n\} : \text{indegree}(v) > 1\}$, this amounts to the following representation.

$$
\begin{aligned}
&((\text{regpath}(v_0), \text{offset}(v_0)),\\
&\ \{\text{regpath}(v_i) \in p : v_{i-1} \in V_{\text{split}} \lor v_i \in V_{\text{merge}}\}),\\
&\ (\text{regpath}(v_n), \text{offset}(v_n))
\end{aligned}
$$

1.6 Multipath Intervals

We define a *multipath interval* as a set of paths with common start and end nodes. Thus, a multipath interval between the vertices v_s and v_e is a subset of all the paths between v_s and v_e denoted $S_{\text{path}}(v_s, v_e)$. We limit the focus

to multipath intervals that can be determined uniquely by a subgraph $G_S = (V', E')$ by the function:

$$MP_g(G_S) = \{(v_0, e_1, v_1, \dots, e_n, v_n) \in S_{\text{path}}(v_0, v_n) : v_i \in V', e_i \in E' \quad \forall i \in \{1, \dots, n\}\}$$

Explisit Representation Given a sequential partitioning $P = P_1, P_2, ..., P_N$, such multipath intervals can be defined by a set of region paths given by $R = \{P_i \in P : \exists v \in V' \text{s.t.} \quad v \in P_i\}$. Multipath intervals can then be represented by the set of region paths with the function:

$$MP_r(R, v_s, v_e) = \{(v_0, e_1, v_1, \dots, e_n, v_n) \in S_{\text{path}}(v_s, v_e) : \exists P \in R \text{s.t.} \quad v_i \in P\, \forall i \in \{0, \dots, n\}\}$$

Thus, a multipath interval can be represented by giving the start and end vertex and explicitly specifying the 'allowed' region paths.

Critical Subpaths Representation A less stringent representation of multipath intervals can be achieved by only specifying the critical subpaths that are required in order to uniquely represent the interval. Subpaths can for instance be represented by region paths or intervals. For instance, given a set of critical region paths $R \subset P$, a multipath interval can be determined by the function:

$$\begin{aligned} MP_{\text{critical}}(R, v_s, v_e) = \{&(v_0, e_1, v_1, \dots, e_n, v_n) \in S_{\text{path}}(v_s, v_e) : \\ &\forall r \in R \exists i \in \{1, 2, \dots, n\}\, \text{s.t.} \quad v_i \in r\} \end{aligned}$$

Fuzzy Representation In order to define fuzzy representation, some concepts are needed. Let the *divergents* of path $p = (u_0, f_1, \dots f_m, v_m)$ from $q = (v_0, e_1, \dots, e_n, v_n)$ denoted divergents(p, q) be the set of subpaths $(u_i, f_i, \dots, f_j, u_j)$, $0 \leq i < j \leq m$ of p where only the start and end vertex is in q. Let the divergent-distance between p and q, denoted $D(p, q)$ be the maximal length of a subpath in divergents(p, q) and let the symmetric distance $L_{\text{var}}(p, q)$ be defined as:

$$L_{\text{var}}(p, q) = max(D(p, q), (D(q, p))$$

We can then define the fuzzy multipath interval, given a single path $p = (v_0, e_1, \dots, e_n, v_n)$ and a variation threshold t, as:

$$MP_{\text{var}}(p, t) = \{q \in S_{\text{path}}(v_0, v_n) : L_{\text{var}}(q, p) < t\}$$

2 Experiments

Data referred to can be found in the Github repository, where also instructions on how to run the experiments can be found: https://github.com/uio-cels/genomicgraphcoords/

2.1 Relationship between transcripts on alternative loci and the main path of GRCh38

The alternative loci were connected to the main path using the locations given in grch38_alt_loci.txt. We define flanks as the continuous regions at the start and end of alternative loci that have identical sequence as the main path. These flanks were merged with the main path. Transcript variants from the RefSeq gene table were then translated to this graph, and the transcript variants on alternative loci (alt-loci transcripts) were categorized in two ways, summary and full categorization:

Summary categorization:

- Category A: The alt-loci transcript is in a flank, and there exists a main-path transcript with the same transcript ID in the same region and with almost equal transcript length[1].
- Category B: The alt-loci transcript is not on a flank. There exists a main-path transcript on the parallel part of the main path with the same transcript ID, and almost the same transcript length.
- Category C: The alt-loci transcript is partly on a flank. There exists a main-path transcript with the same transcript ID on the main path with almost equal transcript length and parallel to the alt-loci transcript on the parts that are not on the flank
- Category D: The alt-loci transcript is in a flank, but there does not exist a main-path transcript in the same region.
- Category E: Any of the first 3 categories above, but where the main-path transcript extends to parts that are not parallel to the alt-loci. The alt-loci transcript is then assumed to be cut at the end of the alternative loci.

Full categorisation: We first classified the alt-loci transcript based on whether there was a parallel main-path transcript and whether that transcript had the same transcription length. Table 1 shows the distribution of alt-loci transcripts over the classes for each type of positioning of alt-loci transcript: Flanking region (flank), varying region (var) or both (flank+var).

Of particular interest is the alt-loci transcripts on the flanking regions without a parallel main-path transcript (8) and the ones with only a partial parallel match on the main path (147). These show that the alt-loci transcripts do not necessarily match the main-path transcripts, even in regions with sequence identity.

[1] Defined as difference in combined exon length less than 5 bp

Table 1: Number of alt-loci transcripts in each category of the full categorization

FLANK			
	Not parallel but	equal transcript length:	6
	Partially parallel but	unequal transcript length:	147
	Parallel and	equal transcript length:	690
	No Matches on same strand	:	2
VAR			
	Not parallel and	unequal transcript length:	40
	Not parallel but	equal transcript length:	12
	Partially parallel but	unequal transcript length:	13
	Parallel but	unequal transcript length:	641
	Parallel and	equal transcript length:	5007
	No Matches on same strand	:	39
FLANK+VAR			
	Partially parallel but	unequal transcript length:	63
	Parallel but	unequal transcript length:	12
	Parallel and	equal transcript length:	198
	No Matches on same strand	:	1

2.2 Representing transcripts using multipath intervals

The following is a description of how transcripts were analysed using multipath intervals.

A graph was created by connecting alternative loci to the main chromosomes, and merging each alternative loci with the main chrosomes according to the NCBI alternative loci to main path alignments (`ftp://ftp.ncbi.nlm.nih.gov/genomes/all/GCA/000/001/405/GCA_000001405.15_GRCh38/GCA_000001405.15_GRCh38_assembly_structure/`).

Critical subpaths multipath intervals

All RefSeq transcripts were represented as critical subpaths multipath intervals on this graph, using the exons of the transcript as critical subpaths (in this case, subpaths were represented as intervals). A match was defined as two transcripts having identical start and end position and identical critical subpaths (i.e. identical exons). This means that two transcripts can follow different paths through the graph, but will still be identical as long as they have the same start and end position and the same critical subpaths.

Fuzzy multipath intervals

All RefSeq transcripts were represented as fuzzy multipath intervals with threshold 10 (each exon represented as a fuzzy multipath interval and the transcription region represented as a fuzzy multipath interval). Two transcripts match if they can be represented with the same fuzzy multipath intervals.
